# Echocardiographic and ultrasound evaluation of haemodynamic parameters in hypoxic neonates treated with hypothermia: Study protocol

**DOI:** 10.3389/fped.2023.1122738

**Published:** 2023-04-18

**Authors:** Natalia Brunets, Veronika Brunets, Renata Bokiniec

**Affiliations:** ^1^ ŻELAZNA Medical Center Ltd St. Sophia’s Specialist Hospital, Warsaw, Poland; ^2^ Lazarski University Medical Faculty of Medicine, Warsaw, Poland; ^3^ Department of Neonatology and Intensive Care, Medical University of Warsaw, Warsaw, Poland

**Keywords:** hypoxic-ischemic encephalopathy (HIE), hemodynamics, therapeutic hypothermia, newborn, rewarming, myocardial performance

## Abstract

**Background:**

Episodes of ischaemia-hypoxia in the perinatal period as well as the changes in the redistribution of blood may lead to decreased perfusion and ischaemia of the cardiac muscle. Additionally, there is a negative impact from the reduced contractility of the cardiac muscle secondary to acidosis and hypoxia. Therapeutic hypothermia (TH) improves the late effects in moderate and severe cases of hypoxia-ischaemia encephalopathy (HIE). The direct impact of TH on the cardiovascular system includes moderate bradycardia, increased pulmonary vascular resistance (PVR), inferior filling of the left ventricle (LV) and LV stroke volume. The above-mentioned consequences of TH and episodes of HI in the perinatal period are therefore exacerbation of respiratory and circulatory failure. The impact of the warming phase on the cardiovascular system is not well researched and currently few data has been published on this topic. Physiologically, warming increases heart rate, improves cardiac output and increases systemic pressure. The effect of TH and the warming phase on the cardiovascular values has a decisive impact on the metabolism of drugs, including vasopressors/inotropics, which in turn affects the choice of medication and fluid therapy.

**Method:**

The study is a multi-centre, prospective, case-control, observational study. The study will include 100 neonates (50 subjects and 50 controls). Echocardiography and cerebral and abdominal ultrasound will be performed in the first 1/2 days after birth as well as during warming i.e., on day 4/7 of life. In neonatal controls these examinations will be performed for indications other than hypothermia, most frequently because of poor adaptation.

**Ethics and dissemination:**

The Ethics Committee of the Medical University of Warsaw approved the study protocol prior to recruitment (KB 55/2021). Informed consent will be obtained from the carers of the neonates at the time of enrolment. Consent for participation in the study can be withdrawn at any time, without consequences and without obligation to justify the decision. All data will be stored in a secure, password-protected Excel file that is only accessible to researchers involved in the study. Findings will be published in a peer-reviewed journal and disseminated at relevant national and international conferences.

**Clinical Trial Registration:**

NCT05574855.

## 1. Introduction

Perinatal hypoxia in the form of HIE is a frequent cause of cerebral impairment in neonates. HIE occurs in 3–5 of 1,000 neonates in developed countries (1) and is significantly higher in developing countries at 25 of 1,000 neonates (2). Despite developments in medicine, increasing understanding of HIE pathophysiology and progress in neonatal intensive care as well as treatment of moderate or severe perinatal asphyxia, HIE continues to be associated with significant mortality and late neurological sequelae (1).

### 1.1. Impact of hypoxia on the cardiovascular system

Transitory hypoxia of the myocardium (and its resultant dysfunction), which may, but not necessarily, present clinically, occurs in two thirds of neonates born with perinatal asphyxia (1). There is no doubt that that this is one of the more frequent cause of circulatory insufficiency. Both an episode of ischaemic hypoxia in the perinatal period and the changes in the distribution of blood may lead to diminished perfusion of the cardiac muscle. An additional but no less important impact on cardiac function is the immaturity of the neonatal myocardium and its reduced contractility secondary to acidosis and hypoxia. Ischaemia and acidosis lead to imbalance in favour of production of endothelin 1, which leads to reduced production of nitric oxide and vasoconstriction of pulmonary vessels and therefore greater PVR which has a detrimental effect on the already impaired right ventricular (RV) function. The weak RV function and increase PVR impair filling and function of the LV and thus, they can affect systemic and cerebral blood flow (2, 3).

### 1.2. The impact of therapeutic hypothermia on the cardiovascular system

TH improves the distant results in cases of moderate and severe HIE and is currently the standard of care for neonates born at or near term (>35 weeks of gestation). The direct effect of TH on the cardiovascular system includes the following:

• Moderate bradycardia resulting from the decreased effect of the parasympathetic system on cardiac function. Indeed, sinus bradycardia leads to reduced stroke volume and decreased requirement for energy by the myocardium (3–5). In turn, administration of inotropes increase metabolic requirements.
• Additionally, TH leads to increased PVR, potentially resulting in a clinical picture of persistent pulmonary hypertension in the neonate (PPHN) or its exacerbation in cases of pre-existing raised PVR (3, 6). In animal studies (7), TH was associated with increased PVR, while an increased risk of PPHN with TH was not found in randomized controlled clinical trials (RCTs) (5, 10).
• The resulting RV dysfunction and reduced stroke capacity of the RV leads to reduced pulmonary venous return and therefore inferior filling and stroke volume of the LV. A consequence of the effects of TH mentioned above and of an episode of HI in the perinatal period is therefore exacerbation of respiratory and circulatory failure.

### 1.3. Impact of the warming process on the cardiovascular system following administration of hypothermia

The impact of the warming phase on the cardiovascular system has not been well documented and currently very little data was published on this topic. Physiologically warming accelerates the heartbeat and improves stroke volume (6), although the mean blood pressure may fall (7) or remain unchanged as a result of lowering of the diastolic component, which in turn affects metabolism and drug clearance, including clearance of cardiovascular medications. The warming phase, following conclusion of hypothermic treatment, affects the selection of further medicinal therapy in terms of vasopressors, inotropes and of fluid therapy. Furthermore, studies have shown that neonates are more at risk of convulsive episodes during the warming phase. In a study of 160 neonates, 9% experienced intra- or periventricular haemorrhage. Neonates require more precise observation in terms of haemodynamic instability during the warming phase (5) (Table 1).

**Table 1:** Impact on the cardiovascular system.

Impact of TH on the cardiovascular system	Impact of the warming phase on the cardiovascular system
*Cerebral redistribution* • Reduced cerebral blood flow (CBF) • Preferential cerebral redistribution of left ventricular output (LVO) • TH causes vasoconstriction *Sinus bradycardia* • Slower repolarisation of the sinoatrial node Increased influence of the parasympathetic system LVO Reduction 60/70%	• Persistent cerebral redistribution • Preferential cerebral redistribution of the LVO • Decreased systemic vascular resistance (SVR) • Mobilisation (“exaggerated”) effect of inotropes • Increased HR • Increased cardiac output (CO)

## 2. Objectives

Haemodynamic changes were assessed by echocardiography doppler in neonates with HIE during TH and the warming phase.

### 2.1. Primary objectives

Assessment of left and right ventricular systolic-diastolic function during therapeutic hypothermia and in the warming phase.

### 2.2. Secondary objectives

• Assessment of cerebral circulation in neonates and controls by Doppler evaluation of blood flow in anterior cerebral artery (ACA) and middle cerebral artery (MCA), and flow in the superior vena cava (SVC) cardiac output (CO)
• Assessment of visceral circulation in neonates and controls by Doppler blood flow evaluation in mesenteric artery (SMA).
• Assessment of renal circulation in neonates and controls by Doppler blood flow evaluation in right renal artery (RRA).

## 3. Methods and analysis

### 3.1. Study design

This is a multicentre, prospective, case-control, observational study. The number of subjects under observation will be 100 neonates (study summery Tables 2, 3). The enrolment, intervention and assessment schedule is presented in Table 4. Initial echocardiography and cerebral and abdominal ultrasonography will be conducted according to physician recommendation on day 1/2 after birth—hypothermia group echo1 (T1) with repeat examination on conclusion of hypothermia, during warming i.e., on day 4/7 of life—hypothermia group echo2 (T2). In view of the need for cooling of the neonate from the first hour of life there is no possibility to conduct theses examinations prior to introducing hypothermia.

**Table 2:** Study summary.

Title	Echocardiographic and Ultrasound Evaluation of Haemodynamic Parameters in Hypoxic Neonates Treated With Hypothermia.	
Methodology	Multi-centre, prospective, case-control, observational study.	
Study duration	The study will take place from June 2021—June 2023	
Study Centre(s)	Neonatal Clinic and Neonatal Intensive Care unit, Warsaw Medical University ul. Karowa 2, 00-315 Warszawa	
	ŻELAZNA Medical Center Ltd St. Sophia's Specialist Hospital Medical Centre Department of Prematurity and Neonatal Pathology	
Objectives	**Primary objectives**:	
	Assessment of contraction-relaxation function of the left and right ventricle during therapeutic hypothermia and warming period.	
	**Secondary objectives:**	
	• Assessment of cerebral circulation in neonatal study and control subjects by doppler assessment of blood flow in the anterior cerebral artery (ACA) and medium cerebral artery (MCA), and flow through the superior vena cava cardiac output (SVC(CO))	
	• Assessment of visceral circulation in neonatal study and control subjects by doppler assessment of blood flow in the mesenteric artery (SMA)	
	• Assessment of renal circulation in neonatal study and control subjects by doppler assessment of blood flow in the right renal artery (RRA)	
Number of Subjects	100 neonates (50 subjects and 50 controls).	
Diagnosis and Main Inclusion Criteria	Inclusion criteria (subjects)	Exclusion criteria
	• Neonates aged ≥35 weeks of gestation with an episode of perinatal hypoxia	• Absence of parental or guardian consent for participation in the study
	• Neonates eligible for hypothermia treatment according to the Standa • ds of Medical Care for the Neonates in Poland (Table 5)	• Congenital cardiac abnormalities
		• Genetic abnormalities
		• SGA < 10 centiles
ClinicalTrials.gov ID:	NCT05574855	

**Table 3:** Strong sides and limitations.

**What is known about the subject?**
• TH has a neuroprotective action in neonates with HIE
• Decrease in body temperature together with the negative consequences of episodes of HIE may exacerbate respiratory and circulatory failure
• Physiologically, the warming phase increases heart cardiac activity and increases systemic pressure, however, the precise impact on the cardiovascular system has not been studied. The haemodynamic instability during this phase requires further observation.
**What this study adds?**
• The haemodynamic changes in the circulatory system during TH and in the warming phase in comparison with the neonatal control group will be assessed.
• The impact of TH and the warming phase on the cardiovascular system including cerebral, visceral and renal circulation will be determined.
**Limitations**
• Cardiovascular haemodynamics during TH may also be disturbed by the administration of inotropic, anticonvulsant sedative agents as well as mechanical ventilation or thrombotic changes.
• The specificity of the transient circulation (presence of patent ductus arteriosus, patent foramen ovale) may interfere with some echocardiographic measurements, such as CO (Cardiac output) for LV (left ventricle) and RV (right ventricle).

**Table 4:** Proposed schedule of enrolment.

Study plan	Birth	1–2 days	3–7 days	0–30 days*
Anthropometry	X			
Medical history collection	X			X
Enrolment	X			
Eligibility assessment		X		
Medication (inotropics, sedatives, anticonvulsants)				X
Ultrasound of brain				X
MRI brain				X
Echocardiography		X^1^	X^2^	

X^1^–1 echo for group T (T1), X^2^–2 echo for group T (T2) and echo in group *N* (*N*).

* Entire observation period.

**Figure 1:**
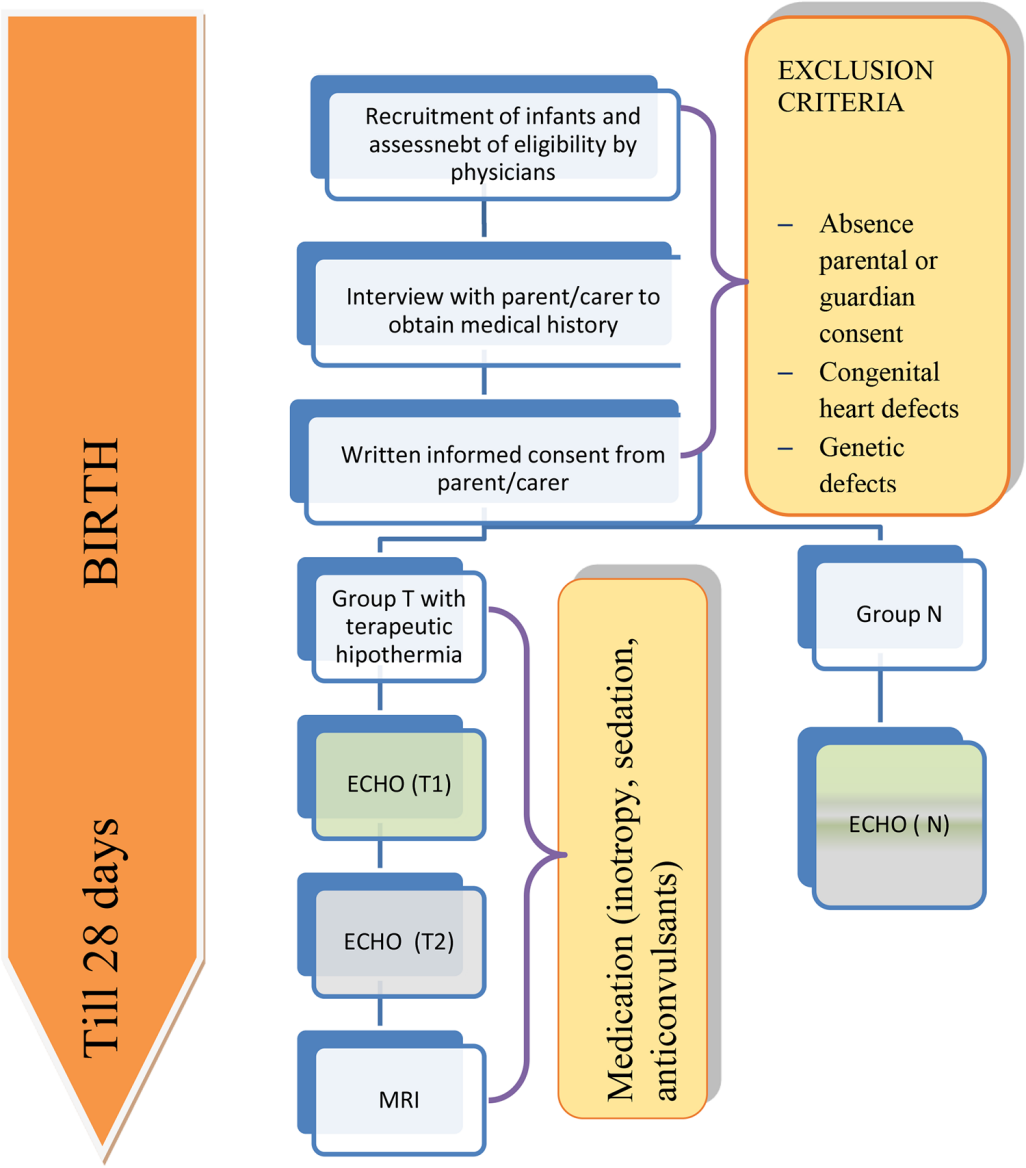
Proposed study protocol.

Images and clips from examinations conducted for indications, other than hypothermia, most frequently because of difficult adaptation, will be used for neonates in the control group (*N*). The study will take place in the period June 2021–June 2023. The proposed protocol of the study is shown in Figure 1.

### 3.2. Study settings

Neonates will be enrolled from patients in the Neonatal Clinic and Neonatal Intensive Care unit, Warsaw Medical University ul. Karowa 2, 00-315 Warszawa and the ŻELAZNA Medical Center Ltd. St. Sophia's Specialist Hospital Medical Centre Department of Prematurity and Neonatal Pathology.

### 3.3. Study population

The study will include 100 neonates (50 subjects and 50 controls).

• Neonates with gestational age ≥35, who experienced an episode of perinatal ischaemia and who were qualified for hypothermia treatment according to the Standards of Medical care of Neonates in Poland will be enrolled to the subject group (T) (Table 5)
• Healthy, term neonates, who underwent echocardiography on days 3–7 of life (after closure of ductus arteriosus, or with trace, insignificant haemodynamic ductus arteriosus) for reasons such as difficult adaptation, gestational diabetes of the mother etc.

**Table 5:** Indications for hypothermic treatment according to the standards of medical care of neonates in Poland 2021 (3).

Indications for TH		
Criteria Group A. Neonates born at ≥35 weeks of gestation, who experienced a period of hypoxia and at at least one of the factors in each of the sub-groups		Group B-neurological criteria. Neonates fullfilling the criteria of group with A with fluctuating states of consciousness who has at least one of the following factors:
Sub-group 1	Sub-group 2	– Hypotonia – Abnormal response to stimuli, including abnormal occulomoor or pupillary reflexes – Absent or weak sucking reflex – Clinically confirmed convulsions
– Apgar score ≤5 week 1, 3, 5, 10 min after birth – Continuous resuscitation with tracheal or mask withinn 10 min after birth	– Cord blood or blood pH within 1 h of life ≤7, however, with specicif assesment of pH results in the region of 7.01–7.15 – A BE base deficiency of at least 16 mmol/l cord blood or other samoles samples within 60 min after birth.	

Enrolment of patients will be based on patient medical records. Physicians responsible for recruitment of patients will gather additional information associated with the inclusion and exclusion criteria (Table 6) through detailed history, taking into account the clinical condition of patients and the results of tests. The study procedure will be explained to patient's guardians who will also receive an information leaflet. Guardians will then be asked to provide two copies of informed consent. Consent forms and information papers will be provided solely in the Polish language.

**Table 6:** Inclusion and exclusion criteria.

Inclusion criteria (subjects)	Exclusion criteria
• Neonates aged ≥35 weeks of gestation with an episode of perinatal hypoxia • Neonates eligible for hypothermia treatment according to the Standards of Medical Care for Neonates in Poland (Table 5)	• Absence of parental or guardian consent for participation in the study • Congenital heart defects • Genetic defect • SGA < 10 centiles

### 3.4. Echocardiographic examination

All cardiac scans will be performed in standard echocardiographic projections with the aid of a sector probe with high frequency 8–12 MHz on a Philips camera. Echocardiography will be performed with continuous electrocardiography (ECG) recording from an ultrasound apparatus. Heart rate (HR) will be recorded. Doppler examination of the cerebral, renal and visceral circulation is an essential examination to assess haemodynamics and is routinely performed in ill neonates.

The scans will be saved as video recordings on storage media. In order to decrease the duration of patient examination to a minimum of 10–15 min, echocardiographic measurements will be performed on the recorded film clips or in the Image Arena echocardiographic program.

All parameters will be measured during end expiration and three consecutive cardiac cycles will be measured and averaged for each measurement.

LV and RV systolic and diastolic function on cardiac echocardiography will be assessed using the following imaging options (11–13):

3.4.A.

Pulse Doppler, M-mode, Tissue Doppler (TDI) (14). The examined parameters will also be determined by the tissue efficiency index, the so-called Tei-MPI index-Myocardial Performance Index, (in pulse and tissue Doppler option, cardiac output (left ventricular output) CO (LVOT), mitral valve (MV) early diastolic velocity/late diastolic velocity (*E*/*A*) ratio. MV *E*′/*A*′ ratio, SVC (CO), peak systolic velocity (*S*′), *E*′, *A*′, tricuspid valve (TV) early diastolic velocity/late diastolic velocity (*E*/*A*) ratio. TV *E*′/*A*′ ratio, tricuspidal annular plane systolic excursion (TAPSE), mitral annular plane systolic excursion (MAPSE), ductus arterious (DA) evaluation, tricuspidal valve regurgitation (rTV):

3.4.1A.

Two-dimensional (2D) M-mode measurements will be obtained from a parasternal long-axis view. The following measurements will be assessed: aortic diameter (AO) at the level of the valvular annulus, LV internal diameter at end diastole (LVIDd) and at end systole (LVIDs), left atrial size, RV diameter at end diastole (RVDd), and LV posterior wall thickness at end systole (LVPWs) and at end diastole (LVPWd). Fractional shortening (FS) will be calculated (LVIDd − LVIDs)/LVIDd × 100. The ECG will be recorded continuously during the examination.

3.4.2A.

Left ventricular cardiac output will be recorded from an apical 5-chamber view with a pulsed-wave (PW) Doppler with the flow volume measured in the middle of the outflow tract at the level of the ascending aorta. Aortic area will be measured in 2D echocardiography from a parasternal long-axis view. CO_LVOT_ (ml/kg/min) = velocity time integral (VTI) × aortic area × heart rate, The ECG will be recorded continuously during the examination.

3.4.3A.

Superior Vena Cava cardiac input will be recorded from subcostal coronal midsection views with a PW-Doppler with the flow volume measured in the middle of the inflow tract. SVC area will be measured in 2D echocardiography from suprasternal views. CO_svc_ (ml/kg/min) = velocity time integral (VTI) × svc area × heart rate, The ECG will be recorded continuously during the examination.

3.4.4A.

A mitral inflow velocity pattern and tricuspid inflow velocity pattern will be recorded from an apical 4-chamber view with a PW Doppler sample volume positioned at the tips of the mitral leaflets and tricuspid leaflets, respectively. Tricuspid and mitral insufficiencies will be evaluated from the measurement of the spatial distribution of the tricuspid or mitral regurgitation jet using colour Doppler examination in the apical four-chamber view. Mitral incompetence and tricuspid incompetence will be graded as trivial, mild, moderate, and severe.

3.4.5A.

The AcT/RVET ratio will be measured at the parasternal short axis view with a pulsed-wave Doppler sample volume positioned at the tips of pulmonary valve: AcT-acceleration time (time to peak velocity of pulmonary blood flow), and RVET-right ventricular ejection time.

3.4.6A.

PW DTI will be performed using a special software package available on the Philips apparatus. This method is capable of providing measurements of ventricular wall motion velocity by positioning the sample volume at mitral and tricuspid valve annuli. In the apical 4-chambers view, the pulsed Doppler sample volume will be placed at the lateral margin of the mitral annulus and lateral margin of the tricuspid annulus. The systolic velocity (*S*), the early diastolic myocardial velocity (*E*), the late diastolic myocardial velocity (*A*) at the time of atrial contraction, and the ratio of *E*/*A* will be determined. DTI time intervals will be measured from recordings. The intervals (*a*) between the end of the late diastolic annular velocity and the onset of the early diastolic annular velocity will be equal to the sum of the isovolumetric contraction time (ICT), isovolumetric relaxation time (IRT), and ejection time (ET). The ET (*b*) will be measured as the duration of the systolic annular velocity (*S*). The sum of the ICT and IRT will be obtained by subtracting b from a. Then the LV MPI and RV MPI indexes will be calculated as (*a* − *b*)/*b*. The IRT will be measured from the pulsed-wave Doppler tissue recordings as the time interval from the end of the systolic annular velocity to the onset of the early diastolic annular velocity and the ICT will be obtained by subtracting the IRT from (*a* − *b*). The ECG will be recorded continuously during the examination.

3.4.7A.

Pulsed Doppler myocardial perfusion imaging (MPI) for the right ventricle. The tricuspid inflow velocity pattern will be measured from an apical 4-chamber view with a pulsed-wave Doppler sample volume positioned at the tips of the tricuspid leaflets. All parameters will be measured during end expiration and five consecutive cardiac cycles will be measured and averaged for each measurement. Diastolic flow through the tricuspid valve in the apical four-chamber view, including early diastolic (*E*) and late diastolic, i.e., atrial (*A*) waves in m/s, will be measured with calculation of the tricuspid valve *E*:*A* ratio.

RV ET will be measured separately from the parasternal short-axis scan plane with a PW Doppler signal placed at the pulmonary valve annulus in the RV outflow tract. The ECG will be recorded continuously during the examination. The calculation of the Tei index (RVMPI) will be considered meaningful for the RV if the difference in heart rate for the inflow and outflow path is 5 beats/min. The isovolumetric contraction time (ICT), isovolumetric relaxation time (IRT), and ejection time (ET) will be measured, and the MPI calculated using the formula (ICT + IRT)/ET, and calculated as (*a *−* b*)*b*. Doppler echo (clicks) of the opening and closing of the aorta valve (AV) and MV will be used as reference points to estimate the timing of the ejection period.

3.4.8A.

Pulsed Doppler MPI for the left ventricle. The mitral inflow velocity pattern will be recorded from an apical 5-chamber view with a pulsed-wave Doppler sample volume placed below the mitral valve towards the ventricular septum with the PW-Doppler tracing including both the *E*/*A* (positive) and the aortic (negative) blood flow waveforms. Diastolic flow through the mitral valve in the apical five-chamber view, including early diastolic (*E*) and late diastolic, i.e., atrial (*A*) waves in m/s, will be measured with calculation of the valve *E*:*A* ratio. The ECG will be recorded continuously during the examination. The isovolumetric contraction time (ICT), isovolumetric relaxation time (IRT), and ejection time (ET) will be measured, and the MPI will be calculated using the formula: (ICT + IRT)/ET, and calculated as *a* − *b*/*b*.

3.4.B.

Doppler measurements of other organs: resistive index (RI) in RRA, SMA, ACA, MCA (11).

**Figure 2:**
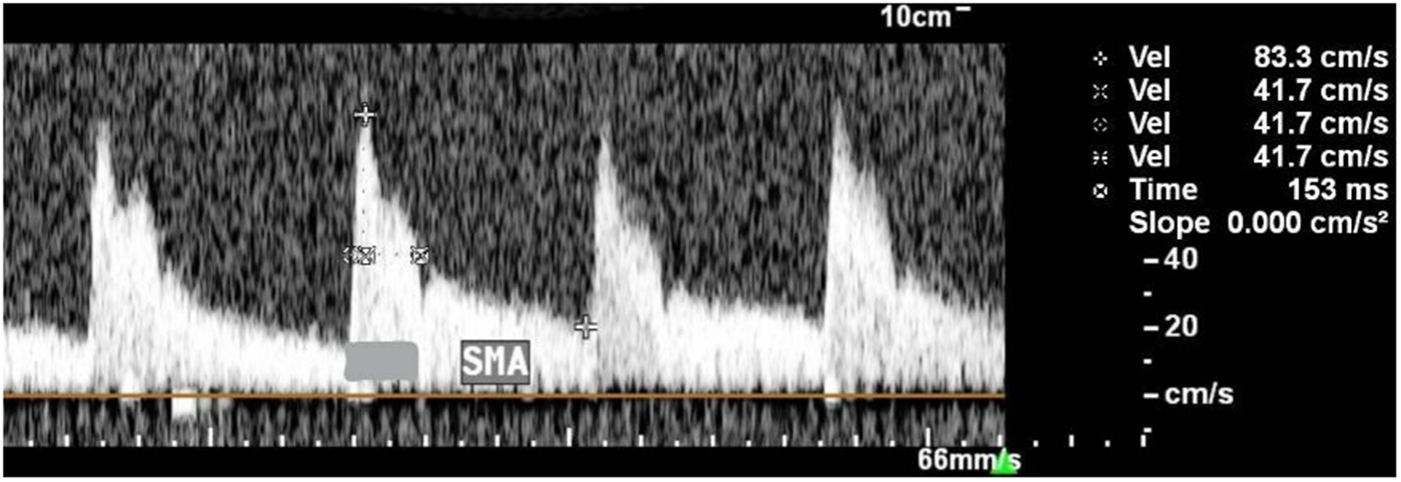
Description of technique for measuring the h-PSV deceleration time in SMA. The PSV was 83.3 cm/s and the h-PSV was 41.7 cm/s. The horizontal dotted line indicates the h-PSV deceleration time (153 ms).

The resistance index has an inverse relationship with blood flow and will be calculated = (PSV − EDV)/PSV. PSV, peak systolic velocity; EDV, end-diastolic velocity. Since bradycardia can interfere with the measurement of blood flow indices: RI and PI, the authors additionally investigated half peak systolic velocity (h-PSV) deceleration time (DT) in ms, which evaluates the time that it takes a single flow velocity waveforms (FVWs) to halve its maximum systolic velocity. A perpendicular line was then drawn from the point of half the maximum velocity to the spectral waveform, which measures the deceleration time in ms (15) Figure 2.

#### 3.4.1B. MCA

The MCA travels laterally and enters the sylvian fissure and is the largest branch of the internal carotid artery supplying the basal ganglia, parietal and temporal lobes. The course of the artery makes it ideal for Doppler assessment. A sectoral transducer 8–12 MHz will be used and placed superior and parallel to the zygomatic arch with the marker directed anteriorly. The colour Doppler will be used, tilting or sliding the probe gently to identify the circle of Willis, with importance to set the colour scale low (10–20 cm/s). The MCA is seen coming directly forward. The PW Doppler cursor will be placed in the proximal portion of the MCA to obtain the velocity profile.

#### 3.4.2B. ACA

The ACA runs in the longitudinal fissure and curves around the genu of corpus callosum supplying the basal ganglia, frontal and parietal lobes. The transducer will be placed in the fontanelle in the midline with the marker directed anteriorly. The ACA will be identified using the colour box, with importance to set the colour scale low (10–20 cm/s). The Power Doppler cursor will be placed in the distal portion of the ACA in order to obtain the velocity profile.

#### 3.4.3B. SMA

The superior mesenteric artery (SMA) is the second artery arising from the abdominal aorta. The SMA supplies nearly the entire small intestine and a substantial part of large intestine. The transducer with a lower frequency of 5–8 MHz will be used, placed below the xiphoid process with the probe marker directed superiorly. A 2D image will be used to find the abdominal aorta and colour Doppler will be used to identify the celiac trunk flow and the SMA flow directed forward, but at an angle. The PW Doppler cursor will be placed in the SMA, sliding and tilting the probe to decrease the angle of insonation and to obtain the velocity profile. The SMA measurement will be performed fasting, prior to the next feeding.

#### 3.4.4B. RRA

A transducer with a lower frequency of 5–8 MHz was used. The transducer will be placed on the flank a few centimetres above the level of the umbilicus, the probe marker directed superiorly. The kidney will be identified in the long axis on 2D imaging. Blood flow from the aorta to the renal hilum will be found using Colour Doppler and the PW Doppler cursor will be placed in the RA prior to the point in which it branches in the renal hilum, sliding and tilting the probe to decrease the angle of insonation and to obtain the velocity profile.

In order to assess the repeatability of the measurements, a pairwise comparison will be made between the results of 2 series of measurements performed by one researcher (N.B.) (intra-observer variability) and two researchers (N.B and R.B) (interobserver variability). On the basis of these series of measurements, the value of the Pearson linear correlation coefficient (*R*) will be determined—as an indirect measure of the impact of random variability on the measurement results and the coefficient of variation (*V*) defined as the quotient of the mean of the squared differences between the pairs of measurements to the product of the standard deviations of both series of measurements. This method of calculating the coefficient of variation provides for taking into account the systematic error component.

### 3.5. The authors classified the parameters into the following groups depending on the evaluation of the function.

• LV function: global LV function-MPI (PD, TDI), systolic function-FS%, LVO, *S*′, MAPSE, diastolic function-*E*:*A* ratio, *E*′:*A*′ ratio measured on MV.
• RV function: global RV function-MPI (PD,TDI), systolic function: *S*′, TAPSE, diastolic function-*E*:*A* ratio, *E*':*A*′ ratio measured on TV**.**
• PPHN: AcT:RVET, TAPSE, incompetence gradient rTV + right atrium pressure
• Systemic Flow (50%): SVCO
• Systemic resistance: h-PSV-half peak systolic velocity, RI-resistance index in SMA, ACA, MCA, RRA

### 3.6. Strengths of the study

To assess the hemodynamics of the circulatory system, the authors also used echocardiographic measurements, the value of which is independent of the presence of DA, PFO, HR, age, body weight, such as the TEI index for RV and LV, and used the tissue Doppler function to assess the myocardium.

An additional advantage of the study is the large size of the study group compared to other publications.

## 4. Treatment or intervention

No study-related interventions are planned. All neonates will be treated according to current guidelines.

## 5. Study outcomes

5.1.

• Assessment of the influence of TH on systolic-diastolic functions of the left and right ventricles in comparison with the control group
• Assessment of the effect of the warming phase on the systolic-diastolic functions of the left and right ventricles in comparison with the control group

5.2.

• Assessment of the influence of TH on the cerebral, visceral and renal circulation in comparison with the control group
• Assessment of the effect of the warming phase on the cerebral, visceral and renal circulation in comparison with the control group

This study assesses the hemodynamic changes by means of echocardiographic Doppler examination in neonates with HIE during TH in the warming phase in comparison with the control group of healthy neonates.

The intention is to assess the impact of TH on the cardiovascular system, including the cerebral, visceral and renal circulation.

TH may have an adverse effect on the cerebral circulation by increasing vascular resistance and loss of adaptation of the cerebral vessels, which in turn can lead to hypoperfusion of vital organs.

The effect of the warming phase on the cardiovascular system by increasing heart rate and improving cardiac output will also be carefully examined. The assumption is that the changes in blood pressure (systolic/diastolic) have a decisive impact on the metabolism of drugs, including vasopressors/inotropics, which in turn affects their choice and fluid therapy.

## 6. Follow-up

There will be no follow-up period.

## 7. Data collection and management

Baseline data will be collected from the hospital electronic database. The following patient data will be collected:

• date and time of birth
• birth weight
• gestational age
• type of delivery
• pH and BE and pCO2 from umbilical cord blood, or blood from the first hour of life
• sex
• Apgar score 1–5–10 min.

The following information will be recorded for each echocardiogram:

• Examination date
• Internal temperature of the neonate in °C
• fraction of inhaled oxygen (FiO2)
• perfusion
• systolic/diastolic/mean arterial pressure
• diuresis, hematocrit, creatinine

The following data will be recorded:

• the need for catecholamines, type of drug, dose or other key drugs: anticonvulsants, nitric oxide (NO), surfactant
• the need for mechanical ventilation (including in the first 72 h of life)
• the need to implement continuous positive airway pressure (CPAP) support within 3 days of life
• occurrence of pneumonia
• occurrence of periventricular bleeding, type of cerebral injury (ultrasound and magnetic resonance imaging (MRI)
• patient's death
• length of hospital stay

The study will use an electronic database.

Entries and corrections to the electronic database will be made solely by the researcher on an ongoing basis. In order to verify the compliance of the data stored in the electronic database with the original source data, access to the source documentation (medical records and ultrasound) will be accessible to the bioethics committee and regulatory authorities. Responsibility for compliance will rest with the principal investigator. The electronic database will conform to the original source data. Periodic checks will be carried out for possible errors and inconsistencies in the data entered.

## 8. Confidentiality

Complete patient and examination information will be stored on a secure, password-protected platform. Only researchers participating in the study will receive a personalized login and password to access information from the study. The statistical team will not have access to confidential data. All relevant medical history data, including copies of ultrasound clips and radiological reports, will be stored separately in electric or paper form in closed folders.

## 9. Statistical analysis

Analysis of sample power and size optimises the use of resources in the study design, increasing the chances of obtaining conclusive results with maximum efficiency. The power is 1 minus the type II error value, i.e., the probability of rejecting *H*_0_ when it is in fact false. The strength and error rate of Type II depend on the specific value of the alternative defined in *H*_1_. The power depends directly and primarily on the following factors:

• the size of the sample used in the study
• the actual size of the effect, i.e., the expected alternative hypothesis
• the adopted significance level

To calculate the power for the *F* test in a design with one factor having *k* levels we will define the hypotheses,

$$H_{0}:\;\mu_{1}=\mu_{2}=\cdots =\mu_{k}$$

and

$$H_{1}:\;\mu_{i}\neq \mu_{j}\;\mathrm{for}\;\mathrm{the}\;\mathrm{levels}\;\mathrm{of}\;\mathrm{the}\;\mathrm{factor}\;i,j\;\mathrm{where}\;i\neq j,$$

where *μ_i_* signifies the mean for level and factor.

The power calculation is based on the *F* test for equality of means in the model (*H*_0_). The *F*-statistic distribution in the case of the null hypothesis is the central *F* distribution, while in the alternative hypothesis it is the non-centralized *F* distribution with the decentrality parameter *λ*. Therefore, power can be defined as the probability that the *F*-statistic follows the decentralized distribution. The exact power is calculated as:

$$\mathrm{Moc}=P(F_{k\text{–}1,N\text{–}k,\lambda }\geq F_{1-\alpha ,k\text{–}1,N\text{–}k})$$

The calculations will be made using the SAS package in Proc Power (rel 15.2).

## 10. Monitoring and harms

The research is observational and non-interventional. Echocardiography and ultrasound examination of the cerebral and renal circulation is an essential diagnostic tool in neonates treated with TH and will not otherwise disrupt the diagnostic and therapeutic process of the neonate. Biological material—will not be collected for the purposes of the study.

## 11. Patient and public involvement

Neither the parents of the patients nor the general public will be involved in developing the questions, measuring outcomes, or developing the study design. There are no plans to involve patients in the recruitment and conduct of the study.

## 12. Implications for practice

The effect of TH and the warming phase on the cardiovascular system, including cerebral, visceral and renal circulation will be determined. The intention is to attempt to show the impact of each phase on the metabolism of drugs, including vasopressor/inotropic drugs, which in turn affects the choice and fluid therapy.

## 13. Ethics and dissemination policy

The Bioethical Committee of the Medical University of Warsaw has approved the study (KB 55/2021). All significant modifications to the protocol will be reported to the committee. Written informed consent will be obtained from all parents/caretakers of infants who are willing to participate in the study. The consent for participation in the study can be withdrawn at any time, without consequences and without an obligation to justify the decision. All deidentified data collected during the trial will be available. These documents will be accessible to anyone who provides a methodologically sound proposal immediately following publication with no end date. We plan to submit our findings to international peer reviewed journals and conferences (paediatric, neonatal).

## 14. Discussion

Asphyxia (A) is characterized by hypoxia and hypercapnia, which occur due to lack of oxygen or ischemia. However, when it lasts longer it can have primarily deleterious effects on the brain, in addition to being associated with impaired organ perfusion, and can result in multiple organ failure.

It also seems likely that hypoxic-ischemic encephalopathy (HIE) is secondary to multi-organ dysfunction, primarily cardiopulmonary failure after asphyxia, and is not solely caused by hypoxia (16, 17). Because severe perinatal asphyxia often causes severe HIE with long-term outcome, most basic and clinical studies in the field of perinatal asphixia have targeted the central nervous system (18, 19).

The number of cardiovascular failures in neonates with asphyxia ranges from 25% to 60% (20, 21) to 78% (22). Hankins et al. (17) confirms in their retrospective study, that in 70% of the newborns studied, HIE was secondary to multiple organ dysfunction.

The direct impact of TH on the cardiovascular system includes moderate bradycardia, increased pulmonary vascular resistance (PVR), inferior filling of the left ventricle (LV) and LV stroke volume (21, 22). The above-mentioned consequences of TH and episodes of HI in the perinatal period are therefore exacerbation of respiratory and circulatory failure. The impact of the warming phase on the cardiovascular system is not well researched and currently few data has been published on this topic. Physiologically, warming increases heart rate, improves cardiac output and increases systemic pressure.

In light of the above written question, and given that one of the goals of therapeutic whole-body hypothermia is to suppress metabolism, is the change we observed simply an adaptive response?

The effect of TH and the warming phase on the cardiovascular values has a decisive impact on the metabolism of drugs, including vasopressors/inotropics, which in turn affects the choice of medication and fluid therapy. Thus, our attention should be shifted from the brain to other organs that may impair the nervous system.

New therapeutic targets must be directed toward normalizing circulation and counteracting the consequences of cardiovascular damage in HIE.

## Data Availability

The original contributions presented in the study are included in the article/Supplementary Material, further inquiries can be directed to the corresponding author.
